# Paraneoplastic leukocytosis and thrombocytosis secondary to IL‐6 elevation in metastatic melanoma

**DOI:** 10.1002/jha2.19

**Published:** 2020-06-25

**Authors:** Nathaniel Wilson, Cristhiam M Rojas‐Hernandez

**Affiliations:** ^1^ Department of Internal Medicine McGovern Medical School The University of Texas Houston Texas USA; ^2^ Section of Benign Hematology The University of Texas MD Anderson Cancer Center Houston Texas USA

A 49‐year‐old man with stage 4 melanoma metastatic to multiple organs, including multifocal osseous lesions, was referred for evaluation of several months of persistent leukocytosis and thrombocytosis. The patient endorsed fatigue from chemotherapy and immunotherapy, but otherwise denied fevers, dyspnea, bleeding, clotting, or history of corticosteroid use. Physical examination revealed tachycardia and tenderness in the right upper quadrant of the abdomen, without lymphadenopathy or splenomegaly.

**FIGURE 1:**
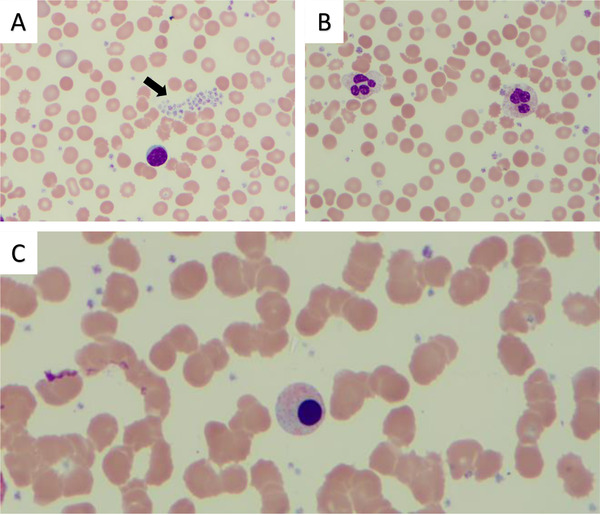
Diagnosing paraneoplastic leukocytosis and thrombocytosis in metastatic melanoma Peripheral blood smears showing (A) reactive thrombocytosis with extensive platelet clumping (black solid arrow). (B) Normal granulocyte morphology and absence of other dysplastic features. (C) Polychromatophilic erythroblast and reticulocytopenia suggesting myelophthisis (Wright‐Giemsa stain). Original magnification ×100

Complete blood count showed a hemoglobin concentration of 10.7 g/dL, MCV 84 fL, and platelet count of 728 × 10^9^/L. White blood cell count was 18.3 × 10^9^/L with 71% neutrophils, 19% lymphocytes, 8% monocytes, 1% eosinophils, and 1% basophils. Peripheral blood smear showed erythroblastosis, with presence of echinocytes and few dacryocytes and fragmented red blood cells, few giant platelets, and extensive platelet clumping, and large mature granular lymphocytes in the setting of malignancy (Figure 1). There was no evidence of circulating blasts. The granulocyte colony stimulating factor level was 36.2 pg/mL, which was within the normal range (RR < 39.1 pg/mL). Interleukin 6 (IL‐6) level was found to be elevated at 10 pg/mL (RR 0‐5 pg/mL).

IL‐6 is a pleiotropic immunomodulatory cytokine that promotes tumor growth by inhibition of apoptosis, and is produced in states of inflammation and malignancy by several types of cells, including melanoma cells.[1] IL‐6 has been shown to upregulate thrombopoietin and platelet production, and has been correlated with hematopoiesis of other cell lines as well.[2] IL‐6 is more commonly associated with reactive thrombosis, and is a rare cause of clonal thrombocytosis.[3] The mechanism of leukocytosis has been described as secondary to IL‐6 inducing neutrophilia through neutrophil mobilization by demarginalization, as well as through accelerating bone marrow release of neutrophils.[4] Elevated serum concentration of IL‐6 has been associated with a worse prognosis in patients with certain types of cancers, including melanoma.[1] Given the peripheral blood smear results, we concluded that this patient's leukocytosis and thrombocytosis were the paraneoplastic result of elevated IL‐6 in the setting of aggressive metastatic melanoma with features of myelophthsis.
